# Myopericytoma in the corpus cavernosum of the penis: A case report of a rare disease

**DOI:** 10.1002/iju5.12583

**Published:** 2023-03-12

**Authors:** Hiroki Oshiro, Yousuke Shimizu, Ryota Nakayasu, Noriaki Utsunomiya, Satsuki Asai, Misa Ishihara, Kimio Hashimoto, Hiroki Katsushima, Sojun Kanamaru

**Affiliations:** ^1^ Department of Urology Kobe City Nishi‐Kobe Medical Center Kobe Japan; ^2^ Department of Pathology Kobe City Nishi‐Kobe Medical Center Kobe Japan

**Keywords:** corpus cavernosum, myopericytoma, penis

## Abstract

**Introduction:**

Myopericytomas usually occur in the extremities of older individuals; however, they also rarely occur in the penis. We report a case of myopericytoma in the corpus cavernosum of the penis and review the relevant literature.

**Case presentation:**

A 76‐year‐old man presented with a slow‐growing painless nodule on the left side of the penis. On physical examination, a non‐tender, 7‐mm mass was palpable. This tumor showed inhomogeneous low signal intensity on T2‐weighted magnetic resonance imaging. The mass was excised and a myopericytoma diagnosed by pathological examination of the operative specimen.

**Conclusion:**

We here report a rare case of myopericytoma in the corpus cavernosum of the penis. To the best of our knowledge, this is the second reported case of a myopericytoma in the penis and the first in the corpus cavernosum of the penis. Clinicians should keep this rare possibility in mind when investigating a mass in the penis.

**Keynote message**

We here present a rare case of a myopericytoma in the corpus cavernosum of the penis. Clinicians should keep this possibility in mind when investigating a mass in the penis.

**Abbreviations & Acronyms**
MRI: magnetic resonance imaging
TUR‐BT: transurethral resection of bladder tumor

## Introduction

Myopericytomas are composed of oval‐to‐spindle‐shaped myoid cells with a tendency to grow concentrically around vessels. They usually occur in the skin and superficial soft tissues of the extremities.^1^ Myopericytomas have been reported in the urinary tract, including the kidney,^2^ bladder,^3^ and glans of the penis^4^; however, to the best of our knowledge, they have not been described in the corpus cavernosum. Most myopericytomas are benign, but some extremely malignant myopericytomas have been reported.^5^

## Case presentation

**Fig. 1:**
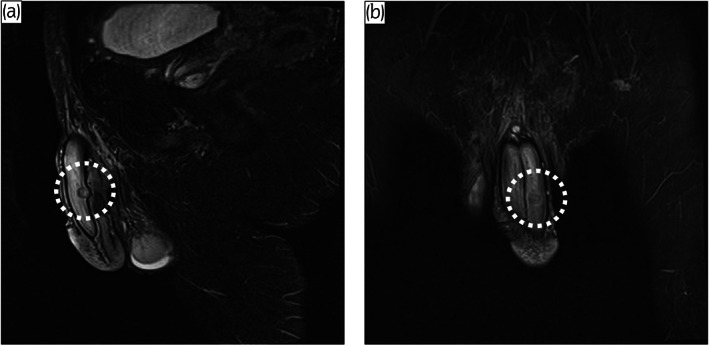
T2‐weighted MRI sagittal (a) and coronal (b) section. The circle shows inhomogeneous low signal intensity.

**Fig. 2:**
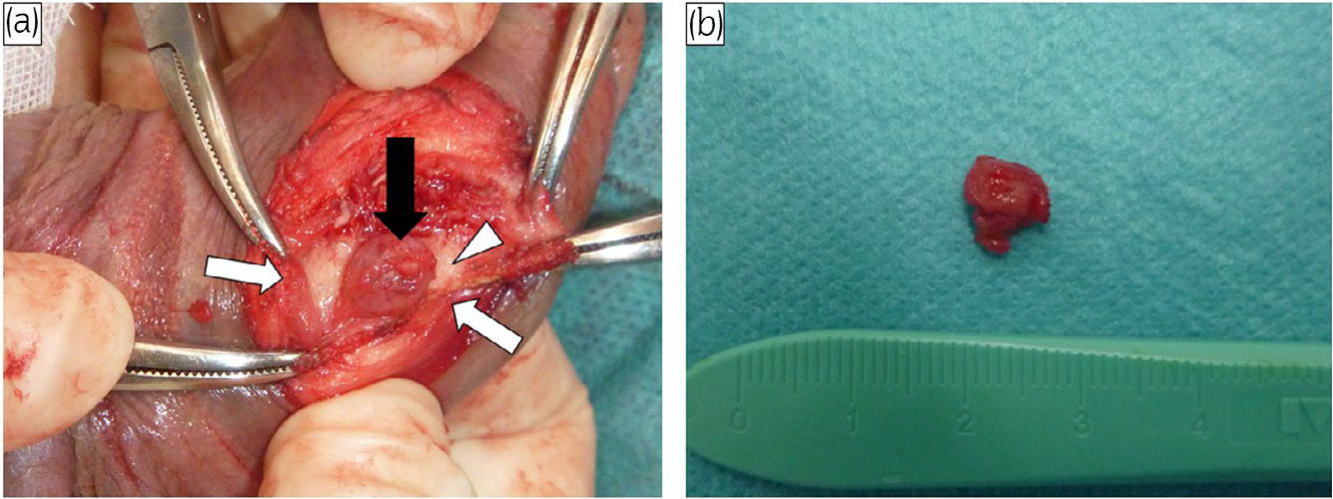
(a) The tumor (black arrow) is arising from the left corpus cavernosum of penis (white arrow) and is partially contact with the tunica albuginea (white arrowhead). (b) Macroscopic appearance of the resected specimen, which measures 7 × 5 mm.

**Fig. 3:**
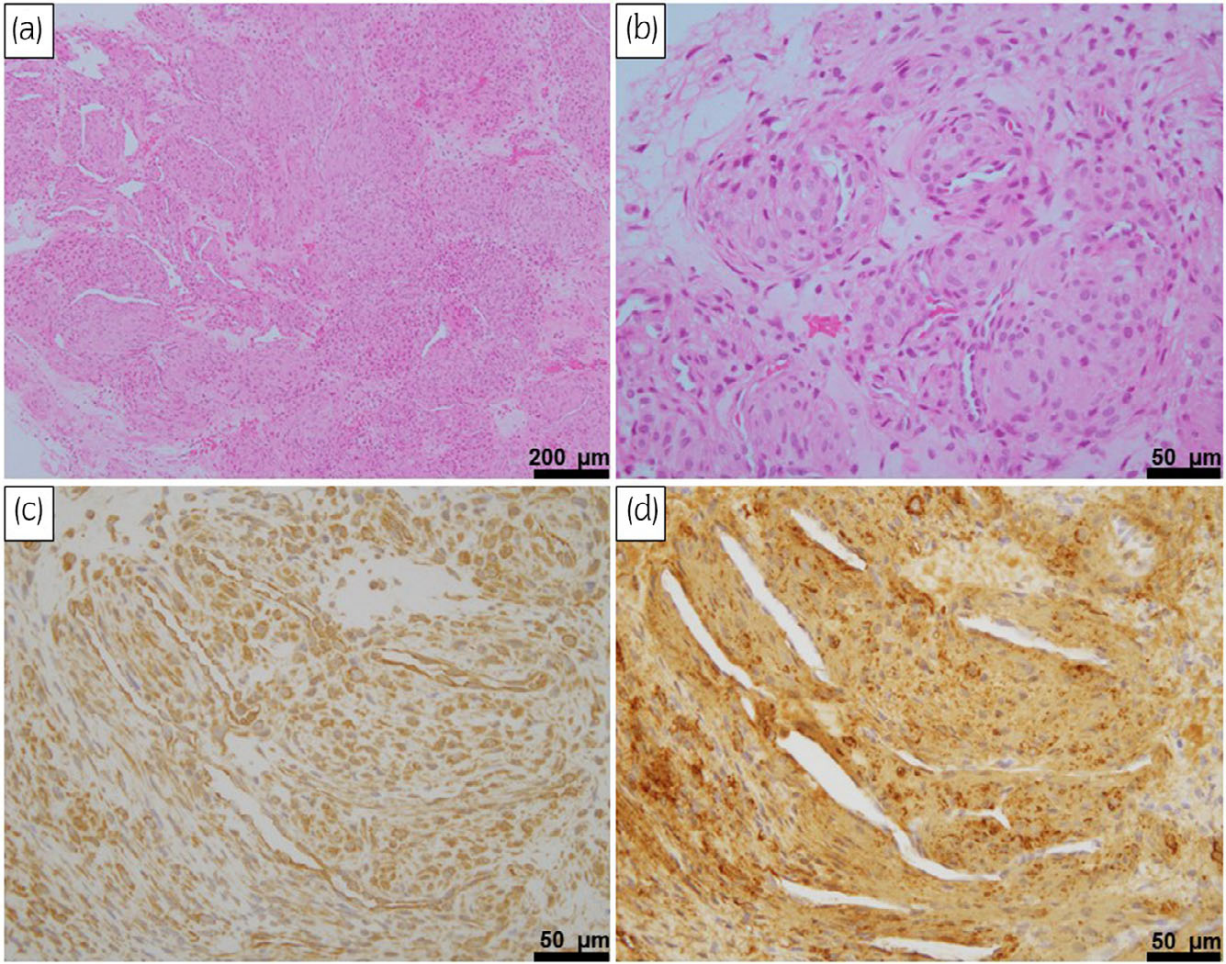
Photomicrographs showing histological findings in the operative specimen: Hematoxylin and eosin staining (a: ×100, b: ×400) and immunohistochemical staining of vimentin (c: ×400) and smooth muscle Actin (d: ×400).

A 76‐year‐old man presented with a small, elastic nodule on the left side of his penis. The mass had been growing for a year and had begun to cause discomfort. On physical examinations, a non‐tender 7‐mm mass was palpable. Ultrasound showed a hypervascular, hyperechoic, well‐circumscribed, solid mass. Magnetic resonance imaging showed a smooth marginated mass with peripheral low signal intensity on T2‐weighted images (Fig. 1). Computed tomography revealed no distant metastases. He had no significant medical history. Considering the patient's symptoms and the possibility of malignancy, we decided that surgical removal was the most appropriate means of alleviating his discomfort and making a pathological diagnosis. Under lumbar anesthesia with the patient lying supine, a sagittal incision was made over the tumor, exposing the corpus cavernosum after incising the dartos and Buck's fascia. The tumor was seen to be arising from the left corpus cavernosum (Fig. 2a) and abutting the tunica albuginea of the corpus cavernosum. It was removed together with part of the tunica albuginea. The total operative time was 51 min, and blood loss was minimal. Macroscopically, the mass was round and solid and measured 7 × 5 mm (Fig. 2b). Microscopically, the tumor was composed of a mixture of smooth muscle cells and prominent vascularity (Fig. 3a). The neoplasm showed multilayered concentric growth around blood vessels and nodules composed of oval‐to‐spindle‐shaped cells (Fig. 3b). It was surrounded by trabeculae comprising collagen, elastic fibers, and irregular cavernous veins, suggesting it had originated in the corpus cavernosum. There was a distinct boundary between the tumor and the tunica albuginea. Immunohistochemical analysis revealed that the tumor was positive for vimentin (Fig. 3c) and smooth muscle actin (Fig. 3d). The patient was diagnosed histologically with myopericytoma and manifested no evidence of malignancy or local recurrences during 18‐month follow‐up.

## Discussion

Myopericytomas were first described by Granter *et al*. in 1998^6^ and first defined by the World Health Organization in 2002.^1^ Myopericytomas, which are often painless, elastic masses, are commonly found in the extremities.^1^ They also occur rarely occur in other sites, such as the lung, heart, brain, liver, and urinary tract.^2^, ^7^ The etiology of myopericytoma has been linked to both trauma and viral infection.^8^, ^9^ These soft tissue neoplasms are believed to arise from pericytes, contractile cells that encircle capillaries and venules and are involved in regulation of blood flow and microvascular dynamics.^1^ To the best of our knowledge, only 17 cases of myopericytomas in the urogenital system have been reported in English (Table 1).^2^, ^3^, ^4^, ^7^, ^10^, ^11^ The median age of onset is 57.5 years (33–78 years) and the 17 cases comprised 10 men (58%) and six women (35%). The reported neoplasms originated in the kidney in 13 cases (76%), bladder in two (12%), and penis in two (12%). Median size was 4 cm (0.7–20 cm). Myopericytomas are often smaller than 2 cm in diameter but tend to be larger when they occur in viscera.^12^ Of the 13 patients with renal myopericytomas, 10 underwent radical nephrectomy and three partial nephrectomy. The two patients with bladder tumors underwent transurethral resection of the tumor and the two with lesions in the penis underwent enucleation. These tumors were histologically identified as myopericytomas and were characterized by perivascular arrangement of plump/spindle‐shaped myoid cells with round or ovoid nuclei, differentiating them from myofibromas, angioleiomyomas, and glomus tumors. All the reported tumors were positive for smooth muscle actin and vimentin on immunohistochemistry studies. Additionally, some were positive for h‐caldesmon (83%), CD34 (40%), and desmin (31%). All reported urinary tract myopericytomas were negative for S‐100 and AE1/3. Their immunohistological features match those of myopericytomas in skin and soft subcutaneous tissues.^12^ None of the reported urogenital system tumors have recurred or metastasized after treatment. Myopericytomas are usually benign; however, rare malignant cases have been reported.^5^, ^13^, ^14^ Malignant myopericytomas have similar histology with the addition of the following characteristics: high cellularity, mitosis, pleomorphism, necrosis, and lymphovascular invasion.^13^ Conradie *et al*. reported that metastases occur in approximately half of patients with malignant myopericytomas.^5^ The liver is the most common site of metastases.^5^, ^14^ No standard treatment has been established; however, complete resection has been performed in all cases with favorable outcomes.^5^

**Table 1:** Summary of 17 reported cases of myopericytoma in urinary tract system including our case

Age (years), median	57.5 (33–78)
Sex, *n* (%)	
Male	10 (58)
Female	6 (35)
Tumor site (%)	
Kidney	13 (76)
Bladder	2 (12)
Penis	2 (12)
Size (cm), median	4 (0.7–20)
Symptoms, *n* (%)	
No symptoms	12 (70)
Abdominal pain	3 (18)
Frank pain	1 (6)
Hematuria	1 (6)
Treatment	
Radial nephrectomy	10 (59)
Partial nephrectomy	3 (18)
TUR‐BT	2 (12)
Enclusion	2 (12)
Immunohistochemical result	
Smooth muscle actin	16 (100)
Caldesmon	10 (83)
Desimin	5 (31)
CD34	6 (40)
S‐100	0 (0)
Vimentin	11 (100)
AE1/3	0 (0)
Recurrence, *n* (%)	
Yes	0 (0)
No	17 (100)
Follow up (month), median	16 (5–66)

Most primary penile tumors arise from penile skin, rarely from the corpus cavernosum.^15^ To the best of our knowledge, there have been only four reports of primary tumors arising from the corpus cavernosum.^15^, ^16^, ^17^, ^18^ One was a benign leiomyoma.^15^ The other three were malignant, comprising a fibrosarcoma,^16^ glomangiosarcoma,^17^ and angiosarcoma.^18^ The prognosis of primary malignancies arising from the corpus cavernosum is poor. Corpus cavernosum tumors are reportedly often metastases, the responsible primary cancers being genitourinary, rectosigmoid colon, lymphoma, and lung.^19^ Diagnoses are often made by biopsy or fine‐needle aspiration performed to differentiate metastatic from primary, as well as benign from malignant, tumors.^19^ We believe that our case is the first report of a myopericytoma in the corpus cavernosum. These differential diagnoses need to be considered in patients with penile cavernous neoplasms.

## Conclusions

We here present a rare case of a myopericytoma in the corpus cavernosum of the penis. To the best of our knowledge, this is the second reported case of myopericytoma in the penis and the first in the corpus cavernosum. Clinicians should keep this rare possibility in mind.

## Author contributions

Hiroki Oshiro: Writing – original draft. Yousuke Shimizu: Supervision. Ryota Nakayasu: Supervision. Noriaki Utsunomiya: Supervision. Satsuki Asai: Supervision. Misa Ishihara: Supervision. Kimio Hashimoto: Supervision. Hiroki Katsushima: Supervision. Sojun Kanamaru: Supervision.

## Conflict of interest

The authors declare no conflicts of interest.

## Approval of the research protocol by an Institutional Reviewer Board

N/A.

## Informed consent

We obtained written informed consent for the publication of details of his case from the patient.

## Registry and the Registration No. of the study/trial

N/A.
